# Enhancement in Power Conversion Efficiency of GaAs Solar Cells by Utilizing Gold Nanostar Film for Light-Trapping

**DOI:** 10.3389/fchem.2019.00137

**Published:** 2019-03-19

**Authors:** Sheng-Qing Zhu, Bin Bian, Yun-Feng Zhu, Jun Yang, Dan Zhang, Lang Feng

**Affiliations:** ^1^School of Materials Engineering, Jiangsu University of Technology, Changzhou, China; ^2^Department of Mechanical and Materials Engineering, University of Western Ontario, London, ON, Canada

**Keywords:** gold nanostars, localized surface plasmon resonances, GaAs solar cells, photocurrent, antireflection

## Abstract

Light trapping, caused by the introduction of metallic nanoparticles, has been demonstrated to enhance photo-absorption in GaAs solar cells. In this study, we successfully synthesized gold nanostar thin film with hot spots and obtained a notable improvement of power conversion efficiency (PCE) in single-junction and three-junction high-performance GaAs solar cells by incorporating the poly (3,4-ethylenedioxythiophene): poly(styrenesulfonate) (PEDOT:PSS) layer, which enables a much stronger light trapping capability and scattering enhancement than conventional metal nanostructures. Increases of 5.2% and 3.94% in short circuit current density (I_sc_) were achieved for single-junction cells and three-junction cells while the enhancement in cell PCE was 3.85 and 2.50%, respectively. The relationship between the optical characteristics, the distribution density of the gold NSs and the performance of GaAs solar cells was systemically investigated.

## Introduction

Since the first research on noble metal nanoparticles (NMNPs) for enhanced photodetectors by Stuart et al. (Stuart and Hall, 1996, 1998), NMNPs have been intensively studied as a potential route to improve the performance of solar cells (Kim et al., 2017; Li et al., 2018). The antenna-like NMNPs with a geometric size in the subwavelength range excite the localized surface plasmon resonances (LSPRs) arising from the collective oscillation of conduction electrons near the particle surface when the electrons are disturbed from their equilibrium positions (Das et al., 2013; Brawley et al., 2017). In previous studies, NMNPs have been demonstrated to serve as local field enhancers or light scattering centers to increase the photogenerated carrier density in various solar cells, with the highest power conversion efficiency (PCE) below 15% (Chen et al., 2012, 2014b; Tan et al., 2012; Park et al., 2013). However, for GaAs solar cells, NMNPs with different geometrical shapes have seldom been compared for their light trapping effects induced in GaAs solar cells, even though they have been widely acknowledged for their capability of photocurrent enhancement (Nakayama et al., 2008; Liu et al., 2011; Wang et al., 2017; Li et al., 2018). Besides, size-controlled nanoparticles were usually fabricated via an AAO template and the thermal annealing process of evaporated Ag on the cell surface, and the PCE of solar cells produced in this way was significantly lower compared to the commercial GaAs solar cells (Nakayama et al., 2008; Liu et al., 2011). These methods are applicable for the rapid, simple and productive preparation of plasmonic NPs with ordinary structures on GaAs solar cells. However, using AAO membrane as a template, coupled with thermal annealing, for NP preparation is quite expensive for commercialization. Producing NPs with other geometric shapes and surface morphologies via this method, such as Au nanostars (NSs) with anisotropic structures and sharp tips, is impossible considering the difficulty of making a proper AAO template, and could not be integrated for stronger plasmonic effects in GaAs solar cells via thermal annealing. Therefore, developing a simple but effective method to incorporate NMNPs into GaAs solar cells is one of the primary challenges to further improving their power conversion efficiency.

In recent years, a functional film, composed of Poly (3, 4-ethylenedioxythiophene): poly (styrenesulfonate) (PEDOT:PSS) and NMNPs, has been widely applied in organic solar cells (Chen et al., 2014a; Ng et al., 2014; Singh et al., 2017) and hybrid Si photovoltaic devices (Xia et al., 2014), serving as the hole transporting path and optical/anti-refection window. Considering the excellent ability of PEDOT:PSS to collect photocurrents and its compatibility with various materials, it could be easily spin-coated on solar cells as thin films with high uniformity. Furthermore, the uniform dispersion of PEDOT:PSS in water could be further mixed with different NMNPs, which are commonly suspended in DI water and could be tuned by adjusting the NMNP solution volume (Fung et al., 2011). A simple method to incorporate NMNPs with special geometric shapes into GaAs solar cells could be thus derived by coating a thin film of PEDOT:PSS/NMNPs onto the cell junctions. In previous works, spherical Ag or Au nanoparticles have been widely reported in various photovoltaic devices (Tan et al., 2012; Zhang et al., 2018). In order to further improve the light-trapping effect, we proposed a different geometry of Au nanoparticles, Au nanostars (NSs) with anisotropic structures and sharp tips, as a good agent to enhance the performance of GaAs solar cells. Due to the significant plasmonic effect, Au NSs function as the field enhancer and improve the performance of solar cells much more significantly than nanoparticles with other shapes (Kozanoglu et al., 2013). On the other hand, the LSPR band of Au NSs can be tuned from visible to the infrared region by manipulating the length of the particle tips to match the light absorption of the photovoltaic layer (Yuan et al., 2012). Due to these novel properties of Au NSs, in this study we report a simple method to synthesize Au NSs at room temperature and pressure by a chemical synthesis process and apply the functional film of PEDOT:PSS decorated with Au NSs directly onto the single-junction (S-J) and three-junction (T-J) GaAs solar cells for cost-effective coating and higher performances. Through the simulation analysis and various characterizations, we investigate the mechanism of power efficiency enhancement and systematically study the influences of particle density on the performance of the GaAs solar cells.

## Experimental Methods

### Preparation of Au Nanostars (Au NSs)

Au NSs were synthesized with the modified seed-mediated method (Sau and Murphy, 2004). First, 8 ml of 0.01 M aqueous HAuCl_4_ solution was added into 190 ml of 0.1 M cetrimonium bromide (CTAB) solution in a glass bottle with gentle stirring. Then, 1.2 ml of 0.01 M AgNO_3_ solution and 1.28 ml of 0.1 M ascorbic acid solution were added in sequence, forming a transparent solution. Finally, 200 μl seed solution of Au NPs with a particle size of 5 nm was added. After the reaction media were gently mixed, the solution was kept undisturbed for 12 h at room temperature. The color of the solution became blue-purple, indicating the formation of Au NSs (Nalbant Esenturk and Hight Walker, 2009). The solution was then centrifuged at 12,000 rpm for 10 min to harvest the Au NSs before washing. The whole centrifugation-washing step was repeated twice and the Au NSs were resuspended into DI water for later spin-coating on the top layer of the GaAs solar cells.

### Preparation of Plasmonic GaAs Solar Cells

**Figure 1:**
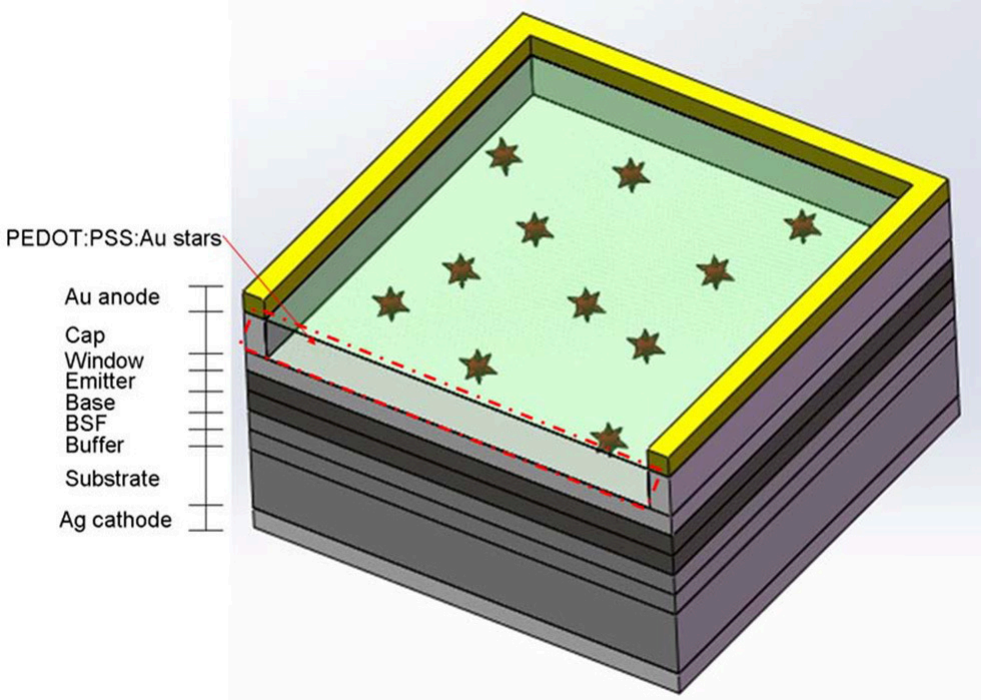
Scheme of plasmonic GaAs solar cell with the PEDOT:PSS film decorated with Au NSs.

The S-J and T-J GaAs solar cells were provided by China Electronics Technology Group Corporation and were fabricated on Ge substrate by a low-pressure metal-organic chemical vapor deposition (MOCVD) system. The structure of S-J GaAs solar cells (2 × 2 cm) was illustrated in Figure 1, consisting of Ag cathode, Ge substrate, buffer layer, back surface field (BSF), base, emitter, window, cap layers, and anode. The thickness of the buffer layer (n-GaAs) was 0.3 μm, on top of which was the 50 nm-thick BSF made of n-type In_0.5_Ga_0.5_P. For the base layer (n-type GaAS), emitter layer (p-type GaAs) and window layer (p-type In_0.5_Ga_0.5_P), the thickness was set at 3.5, 0.5, and 30 nm, respectively. Au anode was deposited on the 60 nm-thick cap layer. It should be noted that there was no anti-reflection layer on the pristine solar cell. The PEDOT:PSS solution with Au NSs was spin-coated on the cell window layer. To examine the impacts of different concentrations of Au NSs bedded in PEDOT:PSS layers, three different mixture ratios of 2.0, 0.5, and 0.125% (V/V) were chosen by adding 200, 50, and 12.5 μl of condensed Au NS solution to 10 ml of PEDOT:PSS, respectively. The top layer of three S-J GaAs solar cells (denoted as pristine cell A, B and C) was spin-coated with the as-prepared mixed solutions at a spinning rate of 5,000 rpm for 45 s and subsequently annealed at 140°C for 10 min. The spin-coated three S-J GaAs solar cells were denoted as plasmonic cell A, B, and C from high to low concentration of Au NSs, respectively. For comparison, another GaAs solar cell (pristine cell D) was spin-coated by pure PEDOT:PSS without Au NSs, to serve as the reference cell, and subsequently annealed at the same condition.

### Characterization

The morphological features of Au nanostars were characterized by Tecnai G2 transmission electron microscope (TEM). Samples for TEM were prepared by dropping ~ 5 μl of nanostar sol onto a carbon-coated copper grid and dried at room temperature. Extinction spectrum was collected with a UV–vis-near-infrared spectrophotometer (UV 2100). The surface morphology of the PEDOT:PSS films decorated with Au NSs was illustrated using a Quanta 400 FEG field emission scanning electron microscope (SEM, FEI Company, USA).

All the solar cells with/without Au NSs were evaluated at 25°C based on the illuminated current density as a function of voltage (J-V), the external quantum efficiency (EQE) and the reflectance characterization. The J-V curves were obtained under the Air Mass 1.5 Global (AM 1.5G) illumination condition (100 mW/cm^2^) by utilizing a solar simulator (Oriel Sol 3A^TM^ class AAA, model 94023A) with a Keithley 2,400 source meter. The EQE was measured with the Bentham PVE 300, while the reflectance spectra of the samples were recorded by using an integrating sphere of the UV–VIS-NIR spectrophotometer (Perkin Elmer, Lambda 1050) for wavelengths ranging from 350 to 900 nm.

## Results and Discussion

**Figure 2:**
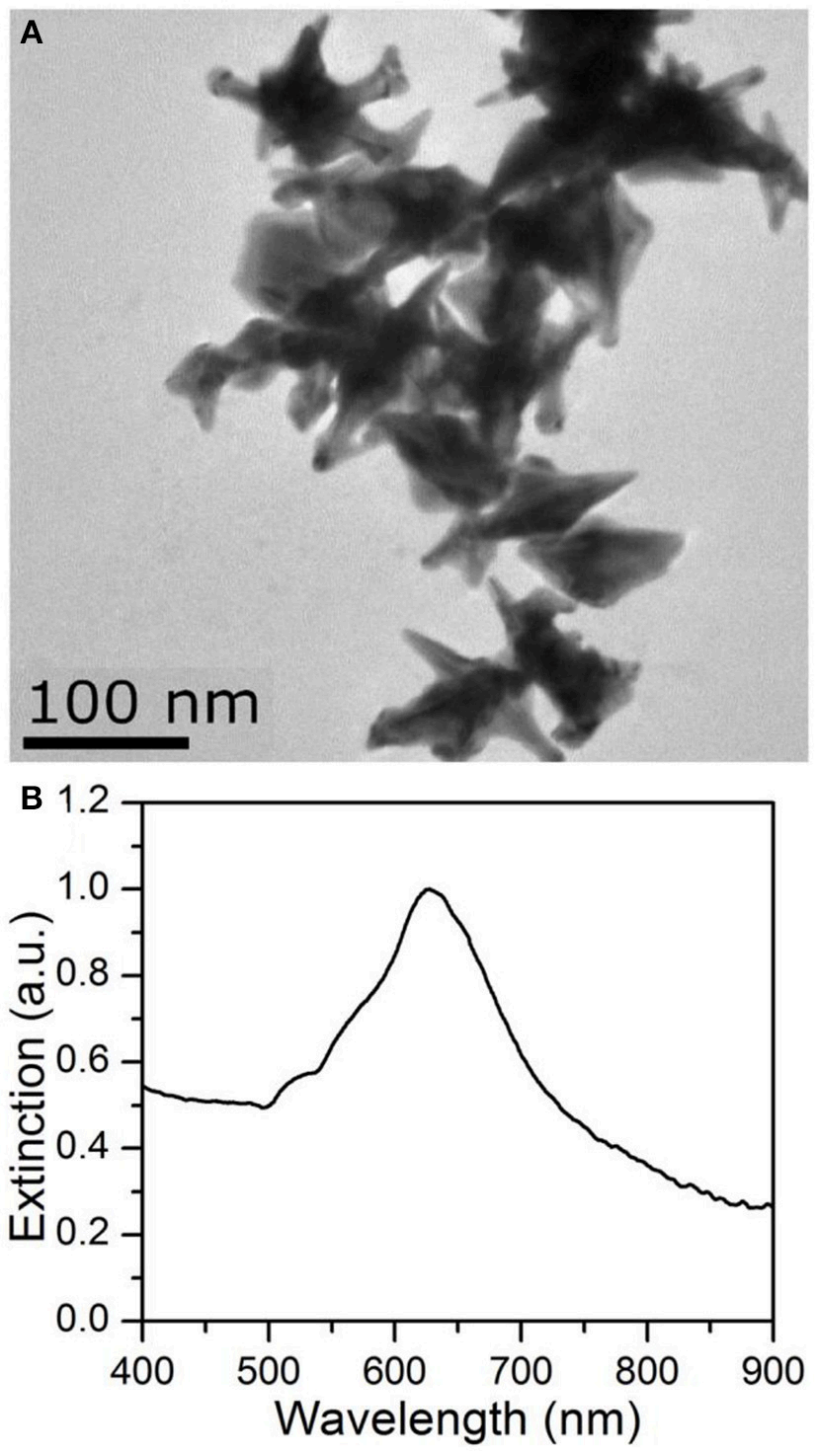
TEM image **(A)** and the extinction spectra **(B)** of Au NSs.

The Au NSs suspended in water solution were characterized by TEM as shown in Figure 2A. The star-shape of Au NPs is clear, with an average size of about 105 nm (tip to tip). By measuring the particle structure, the Au NSs usually possess multiple tips, and the average radius of curvatures at the corner of sharp tips is about 4.6 nm. Figure 2B exhibits the extinction spectra of the Au NS solution, with the LSPR peak near 633 nm. Since LSPR absorption bands are usually dependent on the size, shape, density and local dielectric environment of the nanoparticles, the sharp tips are expected to bring the peak shift in the extinction spectra. The plasmons of the Au NS particle core were reported to have larger frequencies than the tip plasmons, so the introduction of the sharp tip structure onto the core of nanoparticles results in the lower-frequency tip plasmon oscillations of the conduction electrons (Hao et al., 2007). Therefore, sharp tips on the Au NS cores lead to the LSPR peak redshift in comparison to Au spheres (Kumar et al., 2008; Das et al., 2013) and further enhanced the localized electric field intensity in the longer wavelength range. According to previous studies (Li et al., 2011, 2013), the main absorption wavelength of GaAs solar cells is within the range of 600–850nm, which finely covers the LSPR absorption band of the Au NSs. Thus, the integration of these Au NSs is expected to enhance the PCE for the plasmonic GaAs solar cells.

**Figure 3:**
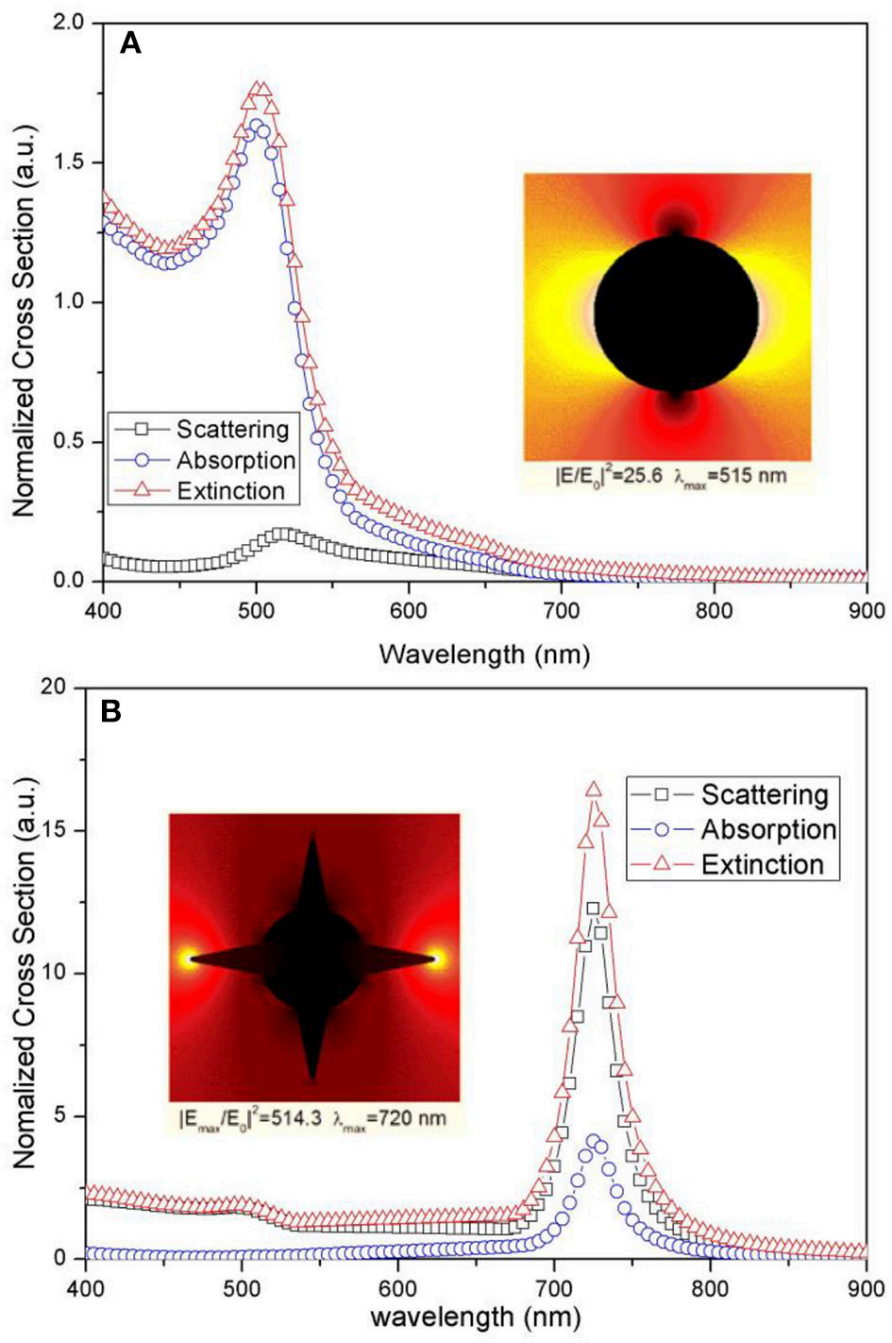
LSPR spectra for Au nanosphere **(A)** and nanostar **(B)**. Inserted images in **(A)** and **(B)** correspond to the cross-sectional views of Au nanosphere and Au nanostar.

To estimate the optical properties and electric-field enhancement obtained from Au NSs, we illustrated the LSPR spectra utilizing the 3D-Finite Element Method for simulation with the numerical program package COMSOL Multiphysics, which has been widely adopted as an effective tool for the modeling of plasmonic devices (Zhang et al., 2011, 2012). In this simulation model, we assume the light illumination on gold nanoparticles to be incident plane waves under TM polarization (TM 01 mode), with wavelengths ranging from 400 to 900 nm. Periodic boundary conditions, which meet actual periodic particle distribution, were set for the simulation of the side boundaries, while boundary conditions with perfectly matched layer (PML) absorbing were used for the top and bottom boundaries of the computational domain. To differentiate Au nanostar from other shapes of Au nanoparticles, Au nanosphere, which is popularly used in plasmonic solar cells (Fung et al., 2011), was also simulated. In this simulation, the environmental condition was set as PEDOT:PSS and the radius of the Au nanosphere and the Au NS core were both set at 30 nm. To remain consistent with the practical shape of the Au NS particle, the radius of the curvature at the corner of NS sharp tip was set at 4.6 nm. As shown in Figures 3A,B, the calculated LSPR for the Au nanosphere and nanostar in PEDOT:PSS peaked at 515 and 720 nm, respectively, indicating the obvious red-shift of the Au NS LSPR peak compared to the Au nanosphere. Several factors accounted for the red-shift of the LSPR peak of Au NSs, such as the size of nanoparticles, the tip numbers and sharpness of the nanostars. Yuan et al. (2012) observed similar phenomena when they did 3D nanostar simulations with different tip numbers and aspect ratios. It was concluded that the sharp tip with high aspect ratios played a significant role in the plasmon shift, which demonstrated the function of sharp tips in the LSPR peak red-shift. As for the minor red-shift change of the Au NS LSPR peak in the simulation compared with Figure 2B, it could be attributed to the refractive difference between the PEDOT:PSS and aqueous environment, the dielectric constant of which affects the LSPR extinction (or scattering) wavelength peak (Willets and Van Duyne, 2007). The optical properties of Au nanosphere and Au nanostar in PEDOT:PSS films were also compared in Figure 3. For comparison purposes, normalized cross-sections at the ordinate were calculated by dividing the scattering, absorption and extinction cross-sections with the geometrical cross-section area of Au nanoparticles. Metal nanoparticles are strong scatters of light at wavelengths near the plasmon resonance, which is due to a collective oscillation of the conduction electrons in the metal. A large scattering cross-section usually leads to a relatively stronger scattering effect, and at the surface plasmon resonance the scattering cross-section can well-exceed the geometrical cross-section of the particle (Catchpole and Polman, 2008). From the simulation results, we notice that Au nanostar has comparatively stronger light scattering effects than Au nanosphere. The normalized scattering cross-section of nanostar reached 12.28 (Figure 3B black line), which was approximately two orders of magnitude higher than the 0.17 from the Au nanosphere (Figure 3A black line). Due to this effect, the optical path length of incident light could be thus increased near the Au NSs and the light absorption would be improved (Atwater and Polman, 2010; Mokkapati and Catchpole, 2012). Furthermore, the scattering cross-section of Au nanostars accounted for a substantial amount of extinction spectra, while the main part of Au nanosphere extinction is absorption. Since the light absorbed by nanoparticles could no longer contribute to the enhancement of photocurrents in solar cells, the larger proportion of scattering light in extinction spectra directly lead to the better utilization of sunlight by photovoltaic materials in GaAs solar cells. Based on the simulation results, the calculated |*E*_*max*_/*E*_0_|^2^ intensity of the Au nanostar could reach as high as 514.3 in the vicinity of the sharp tip area at the resonance wavelength (inserted in Figure 3B), which indicates the great enhancement of photoelectron excitation. However, the Au nanosphere only generates an enhancement of 25.6-fold near the surface at resonant excitation wavelength (inserted in Figure 3A). All these improvements in the optical performances of Au nanostar could be attributed to the strong LSPR effect of the Au nanostar tips.

**Figure 4:**
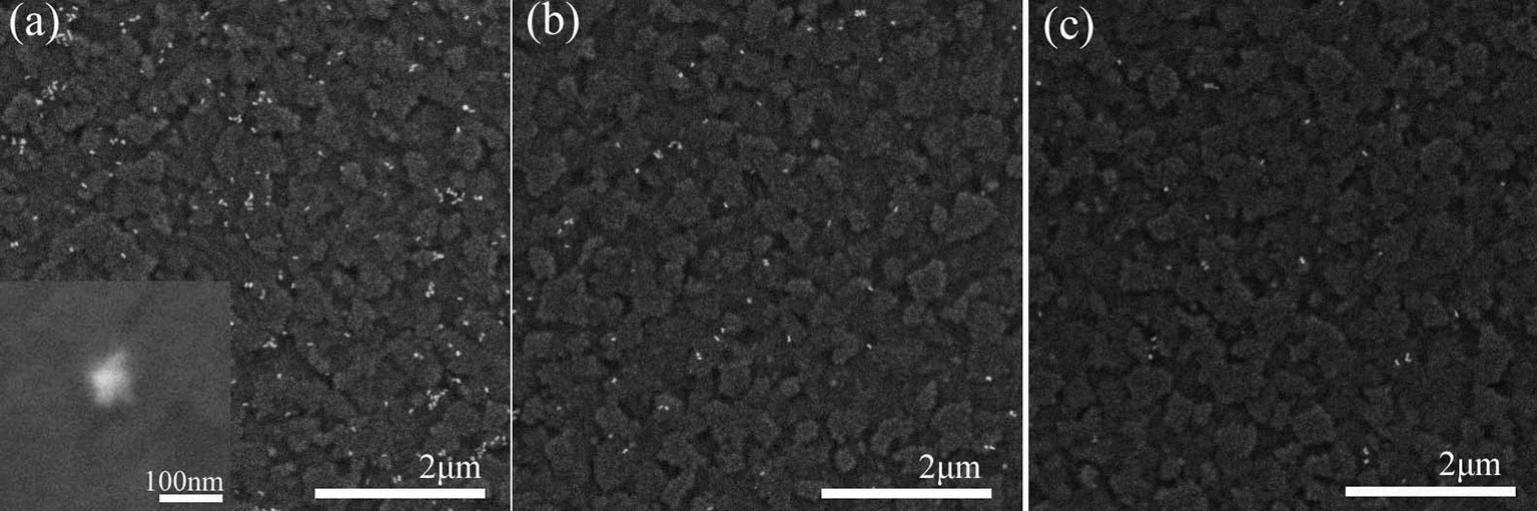
SEM images of the PEDOT:PSS films with different Au NS volume ratio of **(a)** 2.0%, **(b)** 0.5%, and **(c)** 0.125%. The white dots are Au NSs. Inserted image **(a)** correspond to high magnification SEM image of Au nanostar.

Figure 1 exhibits the structure of the high-performance S-J commercial GaAs solar cell. The functional film of the mixed PEDOT:PSS and Au NSs was spin-coated onto the window layer of GaAs solar cells. The resulting thickness of PEDOT:PSS/Au NS thin film is 36 nm, measured by a Dektak stylus profiler. Figures 4a–c represents the SEM images of the functional film of PEDOT:PSS decorated with different Au NS volume concentrations (2.0, 0.5, and 0.125%, respectively) on GaAs solar cells. The SEM image with high magnification (300,000×) inserted in Figure 4a was obtained from the device surface, which clearly demonstrated the presence of Au NSs. As illustrated in the SEM images, Au NSs display excellent dispersion in all the three solid-state PEDOT:PSS films, which indicates the good compatibility of the two materials. The PEDOT:PSS film with higher Au NS concentration distributes uniformly on the window layer and exhibits higher Au NS density. From these three SEM images, we calculated the concentrations of Au NSs (8.08 × 10^12^/m^2^, 2.58 × 10^12^/m^2^, and 7.81 × 10^11^/m^2^, respectively) coated on the surfaces with different volume ratios. Based on the equation of m = ρ•V (in which ρ is the density of materials and V is the volume of the Au NSs), we roughly obtained the weight of the Au NSs with a density of 2.58 × 10^12^/m^2^ on the device's surface (5.7 mg Au/m^2^), assuming the average diameter of Au NSs in this case was ~ 60 nm. The raw material cost (HAuCl_4_, AgNO_3_, CTAB, ascorbic acid, and NaBH_4_ etc.), as low as < 0.85 USD/m^2^,was thus achieved for the fabrication of Au NSs integrated on the solar cell's surface.

**Figure 5:**
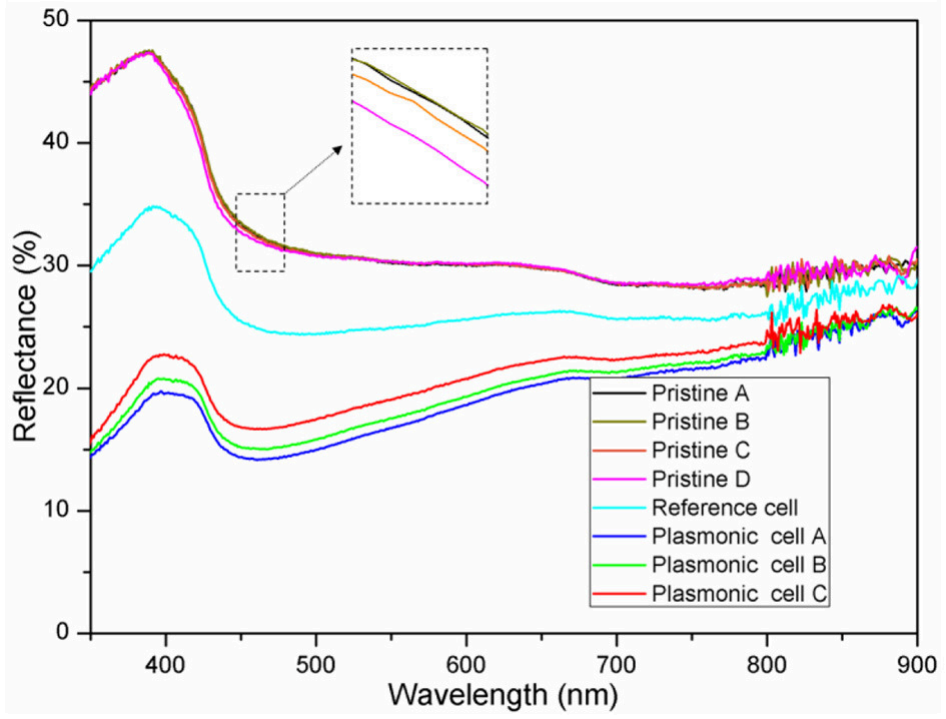
Optical reflectance spectra of the pristine cells A-D, plasmonic cell A-C and pristine cell D decorated with pure PEDOT:PSS.

The optical reflectance spectra of the pristine cells A-D, plasmonic cell A-C and the reference cell with pure PEDOT:PSS are shown in Figure 5. The reflectivity of the four pristine cells exhibited substantial similarity, which clearly demonstrated the excellent repeatability of optical performances of the GaAs solar cells in this experiment. However, the reflectivity of the plasmonic solar cells was obviously reduced along with the increase in Au NS density. The plasmonic cell A with the highest Au NS density (2.0% (V/V)) obtained the lowest reflectivity, indicating the best energy harvesting capability among all GaAs solar cells. The reflectivity reduction along with the increase of Au NS concentration can be largely attributed to the significant LSPR effect of Au NSs and the antireflection effect induced by PEDOT:PSS film (Xia et al., 2014). It is worthwhile to note that the reflectivity of the reference cell decorated with pure PEDOT:PSS also falls in between those of pristine solar cells and plasmonic solar cells, which demonstrated the light adsorption property of the PEDOT:PSS film as mentioned above.

**Figure 6:**
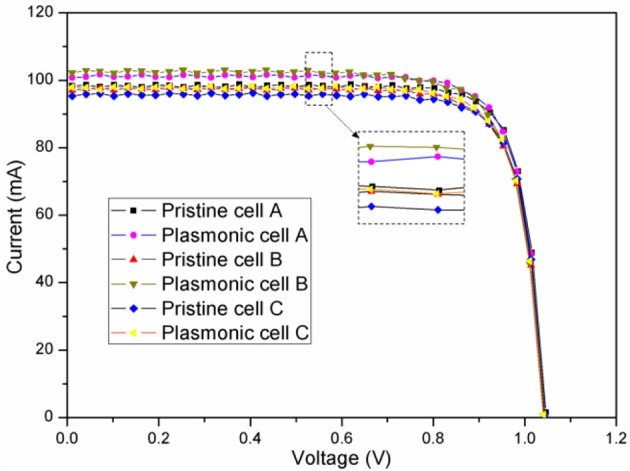
Photovoltaic I-V curves for the S-J GaAs solar cells under AM1.5 illumination.

To determine the effects of the functional films on the photovoltaic performances of S-J GaAs solar cells, current-voltage (I-V) curves and power conversion efficiencies (PCE) were measured under AM1.5 illumination. The photovoltaic properties of the S-J GaAs solar cells are given in Table 1 and Figure 6. The nanocomposite films consisting of PEDOT:PSS and Au NSs had great influences on the photocurrent and PCE of the GaAs cells, in which the best photocurrent enhancement was induced by the 0.5% (V/V) Au NS density. Increases of 5.2 and 3.85% in the short circuit current density (I_sc_) and power conversion efficiency, respectively, were observed for this Au NS concentration. Considering the GaAs solar cell with a power conversion efficiency of 15.31% in this study, an improvement of 3.85% in PCE would bring about an additional 5.89 Watt/m^2^ of illumination condition of AM 1.5. As reported by Lee et al. (2015), the costs for the current S-J GaAs solar cells were about $55.97/W. The power enhancement of 5.89 Watt/m^2^ in this study represents a revenue increase of ~330 USD/m^2^, which outcompetes the cost addition brought by the Au NS coating (< 0.85 USD/m^2^). When the volume ratio of Au NSs to PEDOT:PSS was reduced to 0.125%, the relative enhancement of PCE was restrained to 0.92% compared with the corresponding GaAs solar cell with no Au NS coating. However, for the S-J GaAs solar cell with the highest Au NS density (2%), the relative enhancement of power conversion efficiency also gets weaker (1.39%) compared with the best case. It indicates that the concentration of Au NSs should be optimized for GaAs solar cells, and any further increase in the Au NS concentration has detrimental effects on device performance, which is due to the possibly better performance of electron-hole recombination compared to plasmonic enhancement with excessive Au NSs (Fung et al., 2011).

**Table 1:** I-V characteristics of the single-junction (S-J)/three-junction (T-J) GaAs solar cells.

**Photovoltaic devices**		**V_**oc**_ (V)**	**I_**sc**_ (mA)**	**P_**max**_ (mW)**	**FF**	**Eff (%)**	**Relative enhancement (%)**
S-J cell A	W/o	1.047	98.16	83.75	0.815	15.79	1.39
	Plasmonic	1.044	101.02	84.93	0.805	16.01	
S-J cell B	W/o	1.044	97.29	81.20	0.799	15.31	3.85
	Plasmonic	1.041	102.35	84.40	0.792	15.90	
S-J cell C	W/o	1.044	95.59	80.94	0.811	15.26	0.92
	Plasmonic	1.042	97.72	81.67	0.802	15.40	
T-J cell	W/o	2.690	50.27	114.62	0.848	21.61	2.50
	Plasmonic	2.682	52.25	118.69	0.847	22.15	

**Figure 7:**
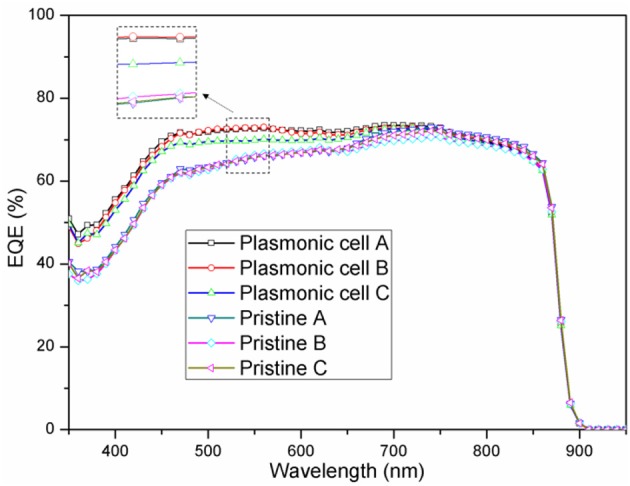
External quantum efficiency (EQE) of pristine cells and plasmonic cell with different Au nanostar densities.

To better understand the mechanism of the improvement in the short circuit current and power conversion efficiency, external quantum efficiency (EQE) spectra were measured as shown in Figure 7. Compared with the pristine cells A-C, the plasmonic cells A-C decorated with PEDOT:PSS/Au NS films exhibited higher EQE values. The plasmonic cells A, B and C with the nanocomposite films obtained an average of 10.5, 12.2, and 9.6% EQE enhancement in the wavelength range of 300–740 nm compared to their corresponding reference cells, which is in excellent agreement with the optical reflectance spectra (Figure 5). Higher short circuit current and power conversion efficiency could be thus attributed to the EQE enhancement based on two possible factors. The first is that Au NSs serve as the sub-wavelength scatters to preferentially forward scatter more light into the GaAs film and lead to an increase in the efficient optical path length in the cell. The other factor for the EQE enhancement comes from the near-field plasmonic effect induced by Au NSs, which could also couple more light into the photovoltaic layer. The absorption of GaAs film has been reported to be proportional to the intensity of the local electric filed (Lagos et al., 2011). Therefore, the entire light trapping and harvesting by the photovoltaic device is enhanced, resulting in enhanced power conversion efficiency.

In previous reports, plasmonic nanoparticles for GaAs solar cells were developed by the “top-down” method, and thus the nanoparticle size, shape and density could not be precisely and systematically controlled, which leads to the restricted LSPR effect and a localized electric field. Our results here demonstrate that the shape of nanoparticles can be precisely selected, and the localized electric field and scattering effects of nanoparticles could be further improved, which prompts an increase in the absorption of incident light. The enhancement in the short circuit current and power conversion efficiency are thus achieved in high-performance GaAs solar cells. In order to prove the wide applicability of this method, we further extended the application of the PEDOT:PSS/Au NS functional film with a volume ratio of 0.5% onto the high-performance three-junction GaAs solar cell. Due to the light absorption enhancement, the current density was improved further from 12.57 to 13.06 mA/cm^2^ (see Table 1). The PCE of the three-junction GaAs solar cell increased from 21.5% before PEDOT:PSS/Au NS film coating to 22.15%, which represents a relative increase of 2.5%. The wide applicability of this functional film coating for the enhanced energy harvesting in both single-junction GaAs solar cells and three-junction cells is thus demonstrated.

## Conclusion

In this study, we investigated the plasmonic effects induced by spin-coated PEDOT:PSS/Au NS films with different Au NS concentrations on the light absorption and power conversion efficiency in single-junction and three-junction GaAs solar cells. The results demonstrated that the excellent PCE improvement can be ascribed to the unique field enhancement and strong scattering effects of Au NSs, which possess multiple “hot spots” on the Au NS surface. Increases of 5.2 and 3.85% in the I_sc_ and PCE, respectively, were observed in the plasmonic GaAs solar cell decorated with PEDOT:PSS/Au NS film with 0.5% (V/V) particle density, due to the stronger localized electronic field, scattering effect and better light absorption. Through a series of measurements including reflectance, I-V characteristics, and EQE testing, the functional film of PEDOT:PSS decorated with Au NSs was proven to be widely applicable for enhancing the performances of various GaAs solar cells. Our study may thus provide a guiding principle of the LSPR effect on high-performance GaAs solar cells.

## Author Contributions

S-QZ and BB conducted the experiment and wrote the manuscript. Y-FZ, LF, and DZ analyzed the data and did the simulation. BB and S-QZ did major work of paper revision and finalization. JY is the supervisor of BB.

### Conflict of Interest Statement

The authors declare that the research was conducted in the absence of any commercial or financial relationships that could be construed as a potential conflict of interest.
